# Nanotoxicology

**DOI:** 10.17795/jjnpp-9982

**Published:** 2013-02-13

**Authors:** Heibatullah Kalantari

**Affiliations:** 1 School of Pharmacy, Ahvaz Jundishapur University of Medical Sciences, Ahvaz, IR Iran

**Keywords:** Nanoparticles, Health, Toxicity

## Editorial

In recent years Nano-structure materials have been attractive to the world of science and technology for their wide spread possibilities in creating new shape and structure associated with materials. The impact of nanotechnology in medicine can mainly be seen in diagnostic methods, drug-release techniques and regenerative medicine. Diagnostic techniques based on the use of nanoparticles offer higher sensitivity and assist the early detection of disease, offering a better prognosis and greater possibilities of successful treatment. The particulate drug delivery system is pharmacokinetic and pharmacodynamics properties of active pharmaceutical ingredient (API). Nanoparticles have attracted a lot of attention of the pharmaceutical scientists in the drug delivery system due to versatility in targeting tissues, accessing deep molecular targets and controlling drug release. Due to its social and economic impacts, nanotechnology has become a focus of public interest.

Understanding the toxicity of Nano-materials is important for human and environmental health. Evaluation the state of knowledge about Nano toxicology is an important step in promoting comprehensive understanding of the health and environmental implications of these new materials. There have been several reviews, reports, and assessments on the current state and/or challenges of determining the toxicology of Nano-materials (1-8).

However, despite the rapid development of nanotechnology, information about the exposure of humans and the environment to Nano-sized particles is very few and the answer of questions about implications of the exposure to nanoparticles for humans and the environment have not yet been sufficiently explained. Therefore the development of technology should be accompanied by a corresponding risk assessment in order to identify and subsequently avoid potential damage. The important questions that should be answered include the following: How stable and persistent are these forms? Do they decompose or agglomerate? Are they soluble in water? Will they interact with other nanoparticles, chemicals, or surfaces? Are they degradable, and how their properties will change during degradation? Nanoparticles can penetrate into live cells. Therefore, they have a potential to accumulate in organisms and thus, also in the food chain and risks associated with this technology have to be paid attention to, thus there is a great need for further research about toxicity of Nano particles.
